# Neonatal LPS Administered Before Sensitization Reduced the Number of Inflammatory Monocytes and Abrogated the Development of OVA-Induced Th2 Allergic Airway Inflammation

**DOI:** 10.3389/fimmu.2021.725906

**Published:** 2021-09-22

**Authors:** Liuchuang Gao, Min Wu, Hangyu Liu, Miao He, Han Jiang, Runshi Shang, Qiangqiang Wang, Zhu Song, Yafei Huang, Junyan Han

**Affiliations:** ^1^Department of Immunology, School of Basic Medicine, Tongji Medical College, Huazhong University of Science and Technology, Wuhan, China; ^2^Department of Obstetrics and Gynecology, Tongji Hospital, Tongji Medical College, Huazhong University of Science and Technology, Wuhan, China; ^3^Department of Pathogen Biology, School of Basic Medicine, Tongji Medical College, Huazhong University of Science and Technology, Wuhan, China; ^4^Institute of Organ Transplantation, Tongji Hospital, Tongji Medical College, Huazhong University of Science and Technology, Wuhan, China

**Keywords:** neonates, time window, LPS, hygiene hypothesis, allergic asthma

## Abstract

It is becoming increasingly clear that environment factors during early life play a pivotal role in the development of allergic asthma. Among these, a traditional farm is one of the strongest protective environments, and the protective effects have been, at least in part, attributed to the high-level exposure to lipopolysaccharide (LPS) on farms. However, the underlying mechanisms remain elusive, especially in ovalbumin (OVA)-induced neonatal allergic asthma model. Here, we used the OVA-induced asthma model in two age groups, neonatal and adult, when mice were first sensitized with peritoneal OVA/alum as neonates and adults, respectively. LPS was injected in the peritoneal cavity before OVA/alum sensitization. The effects of LPS treatment on allergic airway inflammation in the lung and the immune milieu in the peritoneal cavity were determined and compared between these two age groups. We found that LPS treatment abrogated the development of Th2 allergic airway responses in the neonatal group. In the adult group, the ameliorated Th2 allergic responses were accompanied with Th17 responses and neutrophil infiltration upon LPS treatment. We further investigated the immune milieu in the peritoneal cavity to elucidate the underlying mechanisms of this age-dependent difference. Our data show that in neonatal mice, LPS treatment significantly reduced the number of inflammatory monocytes in the peritoneal cavity. In the adult group, LPS treatment shifted the function of these cells which associated with Th1 and Th17 polarization. Our results provide more evidence that immunity in early life is distinct from that in adults, especially in the peritoneal cavity, and emphasize the importance of timing for the intervention of allergic asthma. Our results suggest that LPS treatment during early life is protective for the development of Th2 allergic responses. On the other hand, it might lead to a more severe phenotype of asthma when dampening the Th2 responses in adult mice.

## Introduction

Allergic asthma is characterized by eosinophilic airway inflammation, goblet cell metaplasia, and bronchial hyperreactivity (BHR). The rapid increase in the incidence of allergic disease in the past decades suggests that the changing environmental experiences play a significant role in the etiology of this disease. The hygiene hypothesis proposes that early life exposure with microbe results in protection from development of allergic responses (1). This theory is strongly supported by farmyard studies, which have shown that growing up on a traditional farm protects from the development of allergic asthma (2). On farms, there is a high level of exposure to endotoxin. Indeed, lipopolysaccharide (LPS) is also found in high concentrations in house dust and in stables, mattresses, and raw milk on farms. The protective effects of a high level of environmental LPS have also been demonstrated in these settings (3–5).

Infants have a higher risk of developing allergic asthma than adults. However, the underlying mechanism remains elusive. Studies using early life animal models have confirmed that many facets of immunity in early life are distinct from that in adults (6–8). Regarding the type 2 responses in the lung, the reduction in IL-12 together with the increase in IL-4 in neonates favors Th2 differentiation (9). Recently, it has been demonstrated that the release of IL-33 in the neonatal lungs induced by the first intake of breath is an important contributing factor to the increased susceptibility to allergies in newborns (10, 11). Our group has shown that the deficiency of pDCs in neonates enhances allergic airway inflammation (12).

With the development of novel techniques, such as single-cell RNA-sequencing and multi-omics, both mouse and human studies suggest the existence of a “time window” in early life. This “time window” refers to the key period for immune development and allergen sensitization. Therefore, it serves as the window of opportunity since the immune intervention during this period may affect the susceptibility to allergic disease. In most studies, this “time window” in mice and human refers to the first 3 weeks and the first year of life, respectively (13–15).

Ovalbumin (OVA)-induced allergic asthma model is one of the most commonly used allergic models. In this model, OVA/alum was used for sensitization since this adjuvant provokes strong OVA-specific Th2 responses. As aforementioned, the protective effects of traditional farms have been, at least in part, attributed to the high-level exposure to LPS on farms. Therefore, there have been many studies on the relationship between LPS exposure and the development of allergic asthma. However, given the asthma models used, the timing of LPS treatment, and the concentration of LPS, the results are not completely consistent (16, 17). As a result, whether LPS has a protective effect or is an aggravating factor for allergic asthma is still controversial. In addition, previous studies have mostly been performed in adult mice.

Given the current understanding of the “time window” for allergy intervention, in this study, we focused on the impact of LPS treatment before neonatal sensitization phase by using the neonatal OVA-induced asthma model. We first compared the effects of LPS on OVA-induced allergic airway inflammation. Then we evaluated the milieu of peritoneal cavity to elucidate the underlying mechanism of age-dependent effects of LPS treatment. We found that the Th2 allergic airway responses were ameliorated by LPS treatment in both age groups. However, the underlying mechanisms vary between ages. In neonatal mice, LPS treatment inhibited the recruitment of inflammatory monocytes to the peritoneal cavity. In the adult group, LPS treatment shifted the function of these cells which associated with Th17 polarization and neutrophil infiltration after OVA challenge.

## Materials and Methods

### Mice

BALB/c mice were purchased from Beijing Vital River Laboratory Animal Technology Co., Ltd. (Beijing, China) and were housed under specific pathogen-free conditions. Neonatal mice (both female and male) were used at 7 days of age in this study. Adult mice used in the experiments were female and 7 weeks of age. All experimental procedures performed in this study were approved by the Animal Ethical Committee of Huazhong University of Science and Technology.

### OVA-Induced Asthma Model

The asthma model was performed, as previously described (12). Briefly, neonatal (7 days old) and adult (7 weeks old) mice were sensitized intraperitoneally (i.p.) with OVA (grade V, Sigma) emulsified in aluminum hydroxide (alum, Sigma) on days 0 and 7 (the dose was calculated according to body weight; 20 μg OVA and 4 mg aluminum hydroxide in 100 μl saline for 20 g body weight). From days 14 to 18, these mice were challenged by inhalation of OVA (1% w/v in saline, grade II, Sigma) generated by a jet nebulizer for 30 min. Mice treated with saline were used as control. Twenty-four hours after the last OVA challenge, mice were harvested and the allergic airway inflammation was evaluated.

### LPS Treatment

Neonatal and adult mice were treated i.p. with 1 μg LPS (*Escherichia coli*, Sigma) in 50 μl of saline 1 h before each OVA/alum sensitization. Control mice were treated i.p. with saline.

### Preparation of Bronchoalveolar Lavage Fluid, Lung Single-Cell Suspension, and Peribronchial Lymph Node Cells

Mice were sacrificed and tracheostomized. Lungs were flushed with 1 ml phosphate-buffered saline (PBS). The bronchoalveolar lavage fluid (BALF) was collected. The supernatants were collected for cytokine quantification by ELISA. Cell number in the BALF was evaluated using standard hematologic procedures. In addition, cytospin slides were stained with Wright–Giemsa (Goodbio Technology, Wuhan, China), and differential cell countings in the BALF were performed on the basis of morphological criteria by counting 200 cells under light microscopy.

Lungs were perfused with 5 ml PBS *via* the heart to remove blood cells. Isolated lungs were minced and digested with 1 ml digestion buffer RPMI 1640 containing 5 mg/ml collagenase D (Roche, Carlsbad, CA, USA) and 2 μM EDTA (Ambion, Thermo Fisher Scientific, Waltham, MA, USA) for 30 min at 37°C. Following digestion, 20 μl of 0.5 mM EDTA was added and held for 5 min at 37°C. Lung cells were filtered through a 100-μm cell strainer and red blood cells were lysed with lysing buffer. Lung cell pellets were resuspended in PBS for further experiments.

Peribronchial lymph nodes (PBLNs) were isolated and grinded through a 70-μm cell strainer to prepare single-cell suspensions. To determine cytokine production, PBLN cells were cultured in complete RPMI 1640 medium at 1 × 10^6^ cells/well and restimulated with OVA (grade V, 80 μg/ml) for 72 h. The supernatants were collected and stored at −80°C for further examination.

### Preparation of Peritoneal Cells

To perform peritoneal lavage, neonatal and adult mice were injected i.p. with 1 and 5 ml PBS, respectively. The abdomen was rubbed gently for 2 min, and the peritoneal lavage fluids (PLF) were collected and centrifuged. The supernatants were used for cytokine quantification and cell pellets were resuspended in PBS for further experiments.

### Flow Cytometry and Cell Identification

Lung single-cell suspensions and peritoneal cavity cells were subjected to FACS staining. All cells were blocked with anti-CD16/32 and then stained with fluorescein-labeled antibodies. Lung eosinophils and T cells were stained with fluorescein-labeled antibodies to CD64, Siglec-F, CD3e, and CD4. Lung dendritic cells (DCs) were stained with fluorescein-labeled antibodies to CD11c, MHC-II, CD11b, and CD103. Peritoneal macrophages and granulocytes were stained with fluorescein-labeled antibodies to F4/80, CD11b, Siglec-F, Ly6G, and MHC-II. Peritoneal monocytes were stained with fluorescein-labeled antibodies to CD11b, Ly6C, CD80, CD86, and OX40L. For intracellular staining, cells were stimulated with 50 ng/ml of PMA and 1 μg ionomycin for 5 h at 37°C. After staining of cell surface markers, cells were fixed and permeabilized and stained with IL-5/13/17A and IFN-γ according to the protocols of the manufacturer. All the antibodies were purchased from BD Biosciences (San Diego, CA, USA) or eBioscience (San Diego, CA, USA). Flow cytometry was carried out with the FACSVerse (BD Biosciences, Franklin Lakes, NJ, USA). FlowJo software (Tree Star, Ashland, OR, USA) was used to analyze the data.

### Histology of the Lung

Lung tissues were fixed in 4% paraformaldehyde and embedded in paraffin. Lung sections were stained with hematoxylin–eosin (H&E) and periodic acid Schiff (PAS) before microscopic analysis. The lung inflammation was scored as previously described (18). PAS-stained areas were electronically measured and the mucus index was determined by the following formula: (PAS-stained area/bronchial circumference area) × 100.

### Measurement of Cytokines, Uric Acid, and Serum Ig Levels

The levels of cytokines in the BALF, PBLN cell culture supernatants, and PLF were tested using commercial ELISA kits according to the instructions of the manufacturer. The level of uric acid in the PLF was determined using ELISA kit.

Blood was collected and centrifuged at 4°C, 7,500 rpm for 25 min. The levels of total and OVA-specific IgE and IgG1 in the serum were determined using ELISA kits.

IL-1β, IL-4, IL-5, IL-6, IL-12, TNF-α, IL-17A, IFN-γ, CCL-2, and IgE ELISA kits were from Biolegend (San Diego, CA, USA). The IL-13 ELISA kit was from eBioscience (San Diego, CA, USA), IL-10 and IL-33 ELISA kits were from R&D System (Minneapolis, MN, USA), and IgG1 ELISA kits were from Bethyl (Montgomery, TX, USA). Uric acid ELISA kit was from Eton Bioscience (San Diego, CA, USA). OVA-specific IgE and IgG1 ELISA kits were from Jianglaibio (Shanghai, China). CCL-5 and CCL-11 ELISA kits were from mlbio (Shanghai, China).

### RNA Isolation and Real-Time PCR

To obtain the inflammatory monocytes in the peritoneal cavity, peritoneal lavage was performed on mice 24 h after the first OVA/alum sensitization. PLF cells were harvested and cultured in 2 ml complete DMEM medium containing 10% FBS (Tianhang, Hangzhou, China) for 2 h, and then adherent inflammatory monocytes were obtained (purity > 85%). Total RNA was extracted from 5 × 10^5^ purified inflammatory monocytes using TRIzol^®^ reagent (Invitrogen, Carlsbad, CA, USA). cDNA was synthesized with SuperScript IV reverse transcriptase (Invitrogen) according to the protocols of the manufacturer. Gene expression was analyzed using SYBR™ Premix Ex Taq kit (TaKaRa Bio, Kusatsu, Japan) and the ^△△^Ct method was employed for all real-time PCR analyses. The primers used in this study are listed in **Supplementary Table 1**. All primers were synthesized by Tsingke Biological Technology (Wuhan, China).

### Statistical Analysis

All data were analyzed with GraphPad Prism (version 7.0, GraphPad Software, La Jolla, CA, USA). The differences between groups were calculated using Student’s two-tailed *t-*test and ANOVA with Tukey’s *post-hoc* tests for individual and multiple comparisons, respectively. Data are shown as means ± SD. Differences were considered significant when *P <*0.05: *P < 0.05, **P < 0.01, and ***P < 0.001.

## Results

### Neonatal LPS Treatment Before OVA/Alum Sensitization Abrogated the Development of Th2 Allergic Airway Inflammation

**Figure 1:**
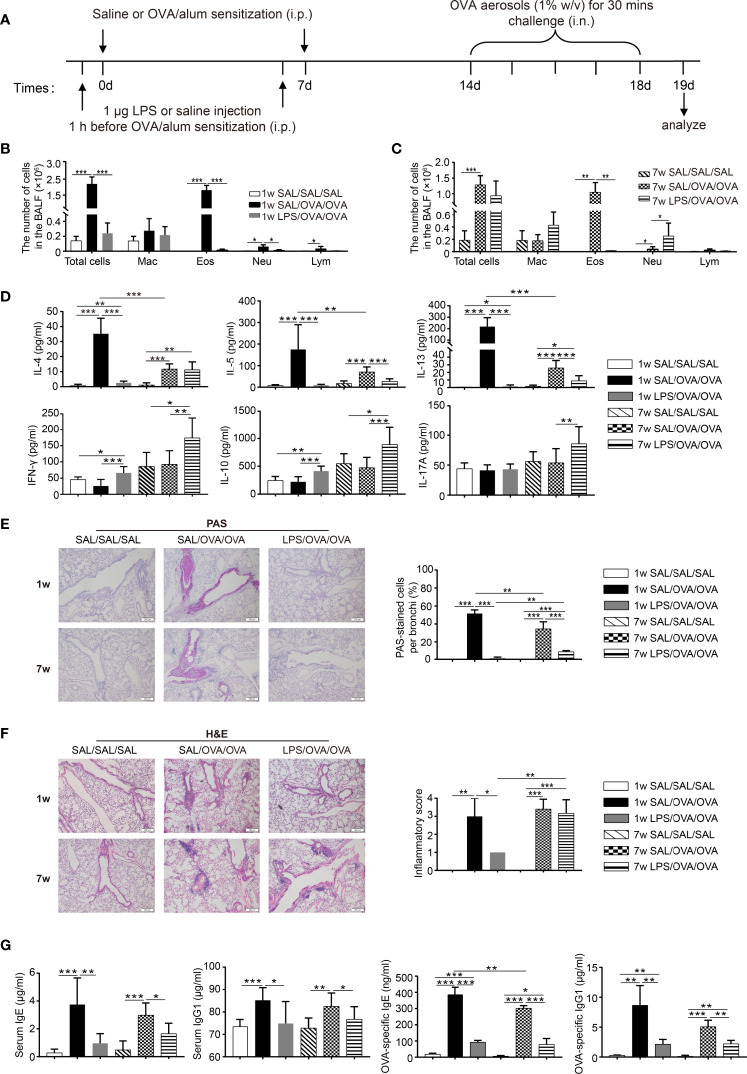
Neonatal lipopolysaccharide (LPS) treatment before ovalbumin (OVA)/alum sensitization abrogated the development of Th2 allergic airway inflammation. **(A)** Schematic of the OVA-induced allergic asthma model. Neonatal (7 days old) and adult (7 weeks old) mice were injected i.p. with 1 μg LPS 1 h before each OVA/alum sensitization and then challenged with OVA *via* airway 7 days after the second sensitization. Twenty-four hours after the last OVA challenge, mice were sacrificed and airway inflammation was evaluated. **(B, C)** Total and differential immune cell counts in the bronchoalveolar lavage fluid (BALF) from neonatal **(B)** and adult **(C)** mice. **(D)** The concentration of cytokines in the BALF was analyzed by ELISA. **(E)** Representative staining and percentage of periodic acid Schiff (PAS)-stained goblet cells. Scale bar = 200 μm. **(F)** Representative section and inflammatory scores of hematoxylin–eosin staining of lung sections. Scale bar = 200 μm. **(G)** The levels of total and OVA-specific IgE and IgG1 in the serum were analyzed by ELISA. Data are representative of three independent experiments (*n* = 3 mice in the SAL/SAL/SAL group, *n* = 5 mice in the other groups). Data are shown as mean ± SD. *P < 0.05, **P < 0.01, ***P < 0.001. Mac, macrophage; Eos, eosinophil; Neu, neutrophil; Lym, lymphocyte; SAL, saline.

LPS has been reported to inhibit the development of OVA-induced allergic asthma in adult mice. However, the effects of LPS on neonatal allergic asthma remain unclear. To compare the effects of LPS treatment before sensitization on the development of allergic airway inflammation in neonates and adults, neonatal (7 days old) and adult BALB/c mice were intraperitoneally (i.p.) sensitized with ovalbumin–aluminum hydroxide (OVA/alum) on days 0 and 7, and then challenged with OVA *via* the airways 7 days later. LPS was given 1 h before each sensitization i.p. (**Figure 1A**). Following OVA sensitization and challenge, both age groups developed Th2 allergic airway inflammation, characterized by airway eosinophilia; elevated levels of IL-4, IL-5, and IL-13 in the BALF; peribronchial and perivascular inflammatory cell infiltration; goblet cell metaplasia; and elevated levels of serum total and OVA-specific IgE and IgG1 (**Figure 1**). When comparing the two age groups, we found that the number of total cells and eosinophils in the BALF and the levels of BALF IL-4/IL-5/IL-13 in the neonatal group were higher than those of the adult group (**Figures 1B–D**).

LPS treatment significantly attenuated OVA-induced Th2 allergic airway inflammation in both age groups. In the BALF, the levels of IL-5 and IL-13 were significantly lower in the LPS-treated groups, while decreased level of IL-4 was only seen in the neonatal group. Upon OVA sensitization and challenge, the level of IFN-γ did not change, while LPS treatment resulted in increased level of IFN-γ in both age groups. The level of IL-10 followed the same trend as IFN-γ. As for IL-17A, only LPS-treated adult mice showed significantly increased level compared with all the other groups (**Figure 1D**). Goblet cell metaplasia was markedly decreased upon LPS treatment (**Figure 1E**), whereas decreased peribronchial and perivascular inflammatory cell infiltration was only seen in the neonatal group (**Figure 1F**). Following OVA sensitization and challenge, the levels of total and OVA-specific IgE and IgG1 in the serum were increased compared with the negative control. LPS treatment suppressed total and OVA-specific IgE and IgG1 production in both age groups (**Figure 1G**). Collectively, these results indicated that LPS treatment significantly alleviated OVA-induced Th2 allergic airway inflammation in both age groups. However, it seems that the protective effect was more pronounced in the neonatal group. On the other hand, LPS treatment in adult mice resulted in an increased level of IL-17A accompanied with the increased number of neutrophils in the BALF (**Figure 1C**), which suggests a trend of Th17 response in the adult group. Taken together, these results indicate that the underlying mechanisms upon LPS treatment may vary between age groups.

### LPS Treatment Before OVA/Alum Sensitization Affected the Immune Responses in the Lung Following OVA Challenge

**Figure 2:**
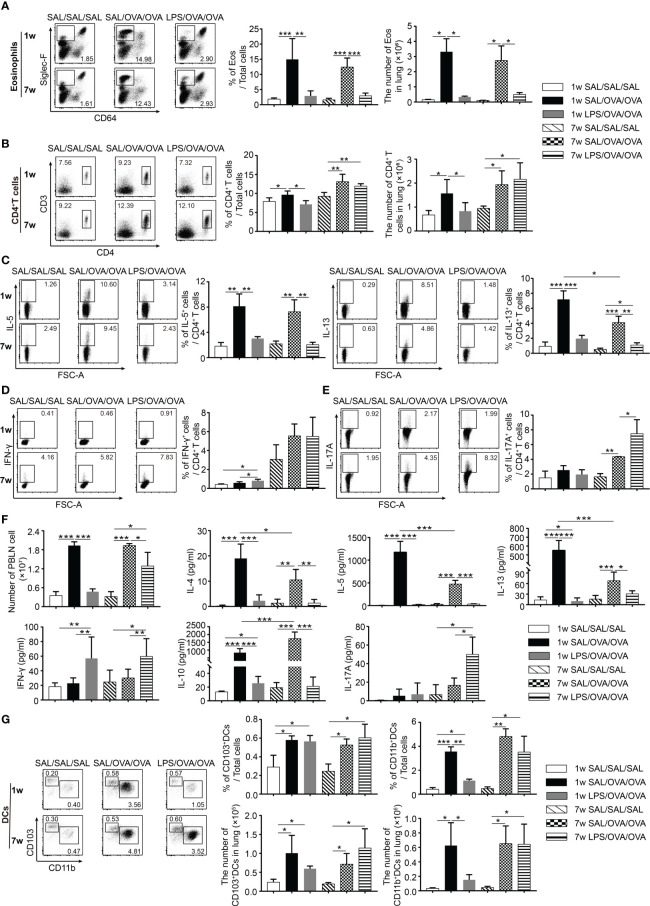
LPS treatment before OVA/alum sensitization affected the immune responses in the lung following OVA challenge. As shown in **Figure 1**, 24 h after the last OVA challenge, lung tissues and PBLNs were dissected and a single-cell suspension was prepared. The percentage and number of eosinophils (CD64^−^Siglec-F^+^) **(A)** and CD4^+^ T cells **(B)** and the percentages of IL-5^+^/IL-13^+^ **(C)**, IFN-γ^+^ **(D)**, and IL-17A^+^ cells **(E)** among CD4^+^ T cells in the lung were assessed by flow cytometry. **(F)** The number of PBLN cells and the levels of cytokines in the culture supernatants from PBLN cells restimulated with OVA for 3 days. **(G)** The percentage and number of lung CD103^+^DCs (CD11c^+^MHC-II^+^CD103^+^CD11b^−^) and CD11b^+^DCs (CD11c^+^MHC-II^+^CD103^−^CD11b^+^) were determined by flow cytometry. Data are representative of three independent experiments (*n* = 3 mice in the SAL/SAL/SAL group, *n* = 5 mice in the other groups). Data are shown as mean ± SD. *P < 0.05, **P < 0.01, ***P < 0.001. PBLN, peribronchial lymph node; SAL, saline.

To explore which type of immune cells in the lung were affected by LPS treatment, a lung single-cell suspension was prepared and analyzed by flow cytometry 24 h after the final OVA challenge (The gating strategy to identify immune cells in the lung is shown in **Supplementary Figures 1A, B**). As shown in **Figure 2A**, the number of eosinophils in the lung was increased following OVA sensitization and challenge, and this number was markedly reduced upon LPS treatment in both age groups. The number of CD4^+^ T cells increased following OVA sensitization and challenge in these two age groups. LPS treatment resulted in a significantly lower number of CD4^+^ T cells only in the neonatal group. Among these cells, the percentages of IL-5^+^CD4^+^ T cells and IL-13^+^CD4^+^ T cells were much lower upon LPS treatment in both age groups (**Figures 2B, C** **)**. The percentages of IFN-γ^+^CD4^+^ T cells and IL-17A^+^CD4^+^ T cells were much lower in the neonatal groups than in the adult groups. LPS treatment increased the percentage of IFN-γ^+^CD4^+^ T cells in the neonatal group but increased the percentage of IL-17A^+^CD4^+^ T cells instead in the adult group (**Figures 2D, E** **)**.

To evaluate the effects of LPS on T-cell differentiation, PBLN cells from each group were harvested 1 day after the final challenge and restimulated with OVA for 72 h. The levels of IL-4, IL-5, IL-13, IL-10, and IL-17A in the culture supernatants were determined. Following OVA sensitization and challenge, the number of PBLN cells was markedly elevated, which declined to baseline level in the neonatal group with LPS treatment. In the adult group, the number was significantly reduced but still higher than that in negative control group (**Figure 2F**). In OVA-sensitized and challenged mice, PBLN cells produced significant amount of Th2 cytokines and IL-10. LPS treatment significantly reduced the levels of all these cytokines and increased the level of IFN-γ. Similarly, as seen in the BALF (**Figure 1D**), the increased IL-17A level was only detected in the adult group (**Figure 2F**). Thus, these results indicated that LPS treatment before sensitization suppressed the Th2 differentiation in the two age groups. Meanwhile, it induced Th1 differentiation in the neonatal group and Th1/Th17 differentiation in the adult group.

DCs have been shown to play a crucial role in maintaining and controlling the adaptive Th2 cell responses to inhaled allergens in the effector phase of asthma (19, 20). We found that the number of CD103^+^DCs and CD11b^+^DCs significantly increased 24 h after the last OVA challenge. LPS-treated mice showed lower number of CD103^+^DCs and CD11b^+^DCs in the neonatal group. However, it was not affected in the adult group (**Figure 2G**).

### Neonatal LPS Treatment Decreased the Number of Inflammatory Monocytes in the Peritoneal Cavity

**Figure 3:**
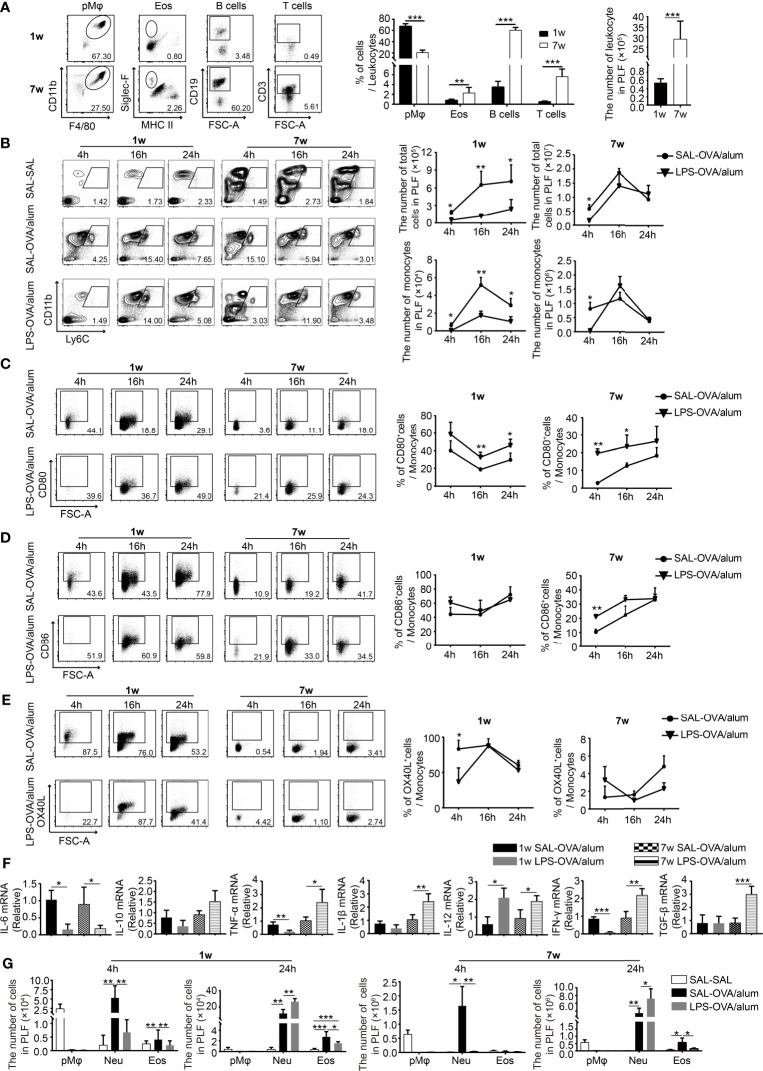
Neonatal LPS treatment decreases the number of inflammatory monocytes in the peritoneal cavity. **(A)** At steady state, the constitution of pMφ (F4/80^hi^CD11b^+^SSC^hi^), eosinophils (Siglec-F^+^MHC-II^−^SSC^hi^), B cells (CD19^+^SSC^low^), and T cells (CD3^+^SSC^low^) in the peritoneal cavity of neonatal and adult mice (*n* = 7 mice per group). **(B–E)** Neonatal and adult mice were injected i.p. with saline or LPS (1 μg) 1 h before OVA/alum sensitization. Four, 16, and 24 h after the first OVA/alum sensitization, the peritoneal lavage fluid (PLF) was collected and the immune cells in the PLF were identified by flow cytometry. **(B)** The numbers of total cells and monocytes (Ly6C^hi^CD11b^+^) in the PLF. The expression of CD80 **(C)**, CD86 **(D)**, and OX40L **(E)** on inflammatory monocytes. **(F)** The peritoneal inflammatory monocytes were purified as described in the *Materials and Methods*. The relative expressions of cytokine genes were determined by real-time PCR. **(G)** The numbers of pMφ, neutrophils (Ly6G^+^CD11b^+^), and eosinophils in the PLF. Data are representative of three independent experiments with *n* = 4 mice per group. Data are shown as mean ± SD. *P < 0.05, **P < 0.01, ***P < 0.001. pMφ, peritoneal macrophage; Neu, neutrophil; Eos, eosinophil; PLF, peritoneal lavage fluid; SAL, saline.

Since LPS treatment was conducted before sensitization, we assumed that LPS exposure before OVA sensitization affected the induction of OVA-specific immune responses, which in turn ameliorate the development of Th2 allergic responses upon OVA challenge. To test this, we first compared the age-dependent difference of the composition of immune cells in the peritoneal cavity at steady state (The gating strategy to identify immune cells in the peritoneal cavity is shown in **Supplementary Figures 1C, D**). The number of leukocytes was higher in adult mice, including macrophages, eosinophils, T cells, and B cells. At steady state, macrophages and B cells are the two predominant cell populations. However, the proportion of them differs significantly between ages: 67.3% ± 4.7% macrophage and 3.5% ± 1.2% B cells in neonatal mice and 20.5% ± 4.3% macrophage and 60.2% ± 5.1% B cells in adult mice. Beside this, the percentages of eosinophils and T cells were higher in adult mice than those in neonatal mice (**Figure 3A**).

To compare the changes of immune cells in different age groups following OVA/alum sensitization and to determine the effects of LPS, we monitored the dynamic changes of immune cells in the peritoneal cavity at 4, 16, and 24 h after the first OVA/alum sensitization. As illustrated in **Figure 3B**, OVA/alum induced significant infiltrations of immune cells in both age groups which peaked at 16 h. It was significantly reduced by LPS treatment in the neonatal group, whereas in the adult group, it was comparable with the control group.

We first analyzed the effects of LPS treatment on inflammatory monocytes given the importance of these cells in the antigen presentation during OVA sensitization. As previously reported (21), the number of inflammatory monocytes increased following OVA/alum injection and peaked at 16 h in both age groups. In neonates, LPS treatment significantly decreased the number of monocytes at each time point. However, in adult mice, it seems that LPS treatment just delayed the recruitment of monocytes, since the number of monocytes was lower at 4 h compared with control, but later after, it was comparable between LPS-treated mice and control mice (**Figure 3B**). We then checked the expression of costimulatory molecules on monocytes (The FMO controls are shown in **Supplementary Figure 1E**). First of all, we found that the expressions of CD80/CD86/OX40L were much higher on neonatal monocytes than on adult counterparts, especially for OX40L, the indicated costimulatory molecule which is important for Th2 cell differentiation. In order to determine whether this phenomenon is the intrinsic character of neonatal monocytes or is caused by the special peritoneal microenvironment of neonatal mice, we compared the phenotypes of peripheral blood monocytes of neonatal and adult mice. We found that the expression of OX40L was low and there was no age difference (**Supplementary Figure 2C**), suggesting that the peritoneal milieu after injection of OVA/alum is highly permissive for Th2 cell differentiation by induction of high expression of OX40L on monocytes, especially in neonatal mice. LPS treatment seemed to increase the expression of CD80, but had no effect on the expression of CD86 and OX40L (**Figures 3C–E**).

To further explore the function of monocytes, these cells were harvested 24 h after the first OVA/alum sensitization. The expression of cytokines was analyzed by real-time PCR. In both age groups, LPS treatment decreased the expression of the IL-6 gene but had no effect on the expression of the IL-10 gene. The expression of all the other genes, including TNF-α, IL-1β, IL-12, IFN-γ, and TGF-β, in the adult group was increased upon LPS treatment, indicating the function shifts from promoting Th2 responses to Th1 responses. It is more complicated in the neonatal group. The expression of TNF-α and IFN-γ was decreased, the expression of IL-12 was increased, whereas the expression of IL-1β and TGF-β was not affected (**Figure 3F**). Taken together, these results indicate that LPS treatment has age-dependent effects on the function of inflammatory monocytes. In neonates, OVA/alum sensitization induced a more pronounced Th2 permissive environment, and LPS treatment significantly reduced the number of monocytes and altered their function.

One of the most prominent cell types found in the peritoneal cavity at steady state is the resident peritoneal macrophages (pMφ) (21, 22). Similar to the phenomenon reported before in adult mice, after OVA/alum sensitization, inflammatory cells were recruited to the peritoneal cavity, along with the dramatic reduction of resident pMφ in both age groups. OVA/alum sensitization induced a significant infiltration of neutrophils in both age groups. LPS seemed to delay the recruitment of neutrophils, while at 24 h, the number of neutrophils was higher upon LPS treatment in both age groups. Eosinophils were recruited into the peritoneal cavity in both age groups after OVA/alum sensitization. LPS-treated mice showed a lower number of eosinophils in both age groups (**Figure 3G**).

### LPS Treatment Before OVA/Alum Sensitization Affected the Microenvironment of the Peritoneal Cavity

To assess the possible mechanisms by which inflammatory cell recruitment and function differed, we measured the levels of alarmins, chemokines, and cytokines in the PLF after the first OVA/alum sensitization.

**Figure 4:**
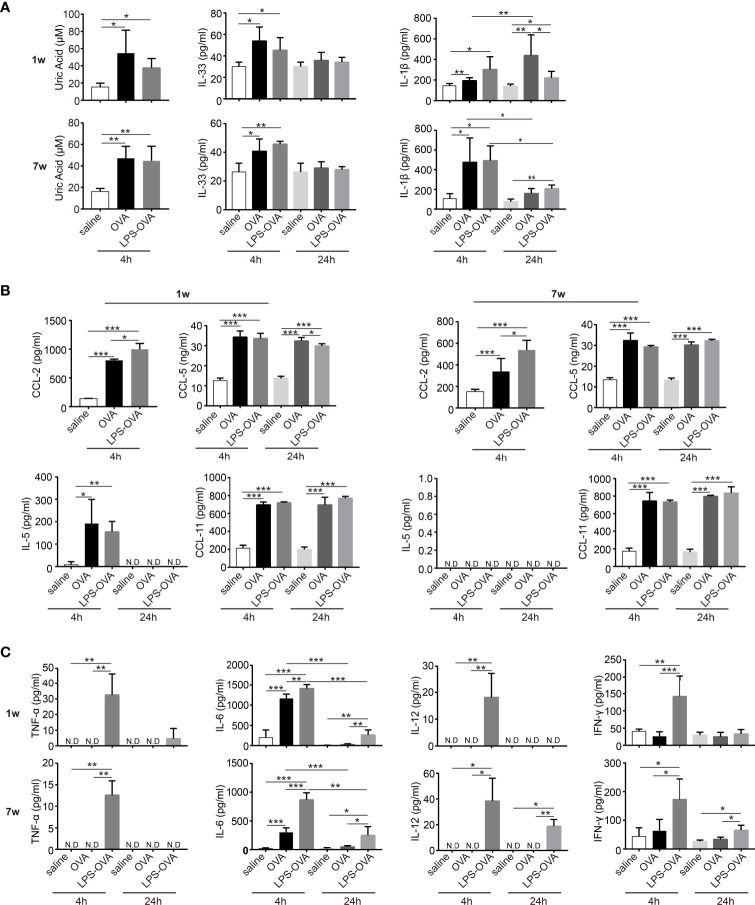
LPS treatment before OVA/alum sensitization affected the microenvironment of peritoneal cavity. **(A–C)** The peritoneal lavage fluids (PLF) from LPS/saline-treated neonatal and adult mice were collected 4 and 24 h after the first OVA/alum sensitization. The levels of alarmins (uric acid, IL-33, and IL-1β) **(A)**, chemokines (CCL-2, CCL-5, IL-5, and CCL-11) **(B)**, and cytokines (TNF-α, IL-6, IL-12, and IFN-γ) **(C)** in the PLF were determined by ELISA. Mouse experiments were performed three times and data are pooled from the analysis of two litters (*n* = 8 mice per group). Data are the means ± SD. *P < 0.05, **P < 0.01, ***P < 0.001. ND, not detected.

Previous studies have shown that the immunopotentiation effect of alum depends on uric acid (18). We found that OVA/alum induced a significant increase in uric acid levels in neonatal mice 4 h after the first sensitization, similar to the response seen in adult mice. However, this was not significantly influenced by LPS treatment in both age groups (**Figure 4A**). Recently, IL-33 has also been indicated in the effect of alum (23, 24), and we then examined the level of IL-33 in the peritoneal cavity. Consistent with previous reports, in the adult group, the production of IL-33 peaked at 4 h after OVA/alum injection and declined to control level at 24 h. LPS treatment showed no effects (**Figure 4A**). In adult mice, OVA/alum induced a rapid increase of IL-1β at 4 h, which fell down at 24 h. LPS-treated mice had a similar trend and comparable level. In neonates, OVA/alum induced IL-1β as well, which peaked at 24 h, and LPS treatment significantly decreased the IL-1β level, even though it was still higher than the negative control (**Figure 4A**).

Based on the changes of constitution of immune cells upon LPS treatment, we then examined the chemokines CCL-2 and CCL-5, which are mainly responsible for the recruitment of monocytes. OVA/alum sensitization induced a rapid production of CCL-2 and CCL-5. Surprisingly, LPS treatment further increased the levels of CCL-2 in both age groups. For CCL-5, LPS treatment resulted in slightly lower levels of CCL-5 in the neonatal group at 24 h. For all the other groups, LPS treatment showed no effect (**Figure 4B**). IL-5 and CCL-11 are important for the recruitment and activation of eosinophils. IL-5 was detected only in the neonatal group right after OVA/alum sensitization and LPS treatment had no effect. The levels of CCL-11 were elevated upon OVA/alum sensitization in both age groups, but still LPS treatment had no effect (**Figure 4B**).

Next, we evaluated the levels of TNF-α, IL-6, IL-12, and IFN-γ in the PLF at 4 and 24 h after each OVA/alum sensitization (**Figure 4C**). In the neonatal group, TNF-α, IL-12, and IFN-γ were not induced at all after OVA/alum injection but significantly increased upon LPS treatment at 4 h. Then the levels of these cytokines dropped to very low levels or under detection limits at 24 h. In the adult group, the patterns of TNF-α, IL-12, and IFN-γ were similar as the neonatal group except that the levels of IL-12 and IFN-γ significantly decreased at 24 h but still higher than the OVA/alum group. IL-6 was induced early after OVA/alum sensitization in both age groups. LPS treatment further increased the level of IL-6. At 24 h, this level was still higher in the LPS-treated group even though it was significantly lower than that at 4 h.

## Discussion

The OVA-induced allergic asthma model is one of the most commonly used allergic models. There have been many reports on the effect of LPS on allergic asthma, but the conclusions are inconsistent. Previous studies have mostly been conducted on adult mice. Given the current understanding of the “time window” (13–15), i.e., it is the period of allergen sensitization and the optimal period for allergy intervention as well, here we focused on the impact of LPS, which was given i.p. at the stage of sensitization. This timing in the neonates falls into the “time window” of OVA sensitization. We then explored the effects of LPS treatment on allergic airway inflammation in the lung and on the microenvironment in the peritoneal cavity and compared these effects in the two age groups.

First of all, characteristic Th2 allergic airway inflammation was successfully induced when neonatal mice were sensitized and challenged with OVA. In addition, the neonatal group showed more severe responses in some ways than the adult group, such as the number of BALF eosinophils, the levels of BALF IL-4/IL-5/IL-13, and the number of PAS^+^ cells in the lung were higher in the neonatal group than those in the adult group. This was further strengthened when the same number of PBLN cells were stimulated with OVA *ex vivo*, and the levels of all Th2 cytokines were higher in the neonatal group. This was consistent with our previous study when using C57BL/6 mouse (12). These results indicate that younger mice are more susceptible to develop type 2 responses.

As in the adult group, LPS treatment significantly attenuated Th2 allergic airway inflammation in the neonatal group. However, the effect of LPS varies with age. It is noteworthy that, in our model, impairment of Th2 responses was more evident in the neonatal group than in the adult group. More importantly, in the neonatal group, the type 2 responses were almost completely diminished by LPS treatment with slightly increased levels of IFN-γ and IL-10 in the BALF. On the other hand, in the adult group, the number of lung neutrophils was increased accompanied by an increased level of IL-17A in the BALF and PBLN cell culture supernatant. In addition, the peribronchial and perivascular infiltration of inflammatory cells was not different from the positive group. These results suggested that LPS treatment appeared to induce a Th17 response when decreasing the type 2 response in the adult group. Recent studies uncovered the involvement of Th17 cells in the pathogenesis of Th2 allergic asthma (25). Accumulating lines of evidence show that the Th2 and Th17 responses are mutually regulated when antibodies to Th2 cytokines enhance the Th17 cell response and neutrophilia and vice versa (26). Here, we observed a similar trend in adult mice that LPS treatment inhibited the Th2 responses and at the same time induced the Th17 responses. As Th17 responses and neutrophilia are always related to a more severe, steroid-resistant asthma, LPS treatment before sensitization might not be beneficial. On the other hand, LPS treatment ablated the development of Th2 responses without induction of Th17 responses in the neonatal group. This suggests that LPS treatment before allergen sensitization in the time window will benefit the patients.

Early life exposure to environmental bacteria may have a long-term effect. In human, it has been demonstrated that the protective effects of early farm exposure can persist into adulthood (27). In animal studies, the protective effects of airway LPS exposure were also shown to last until adulthood (28, 29). Here, in our study, the antigen sensitization and LPS treatment were performed in the first 2 weeks of age, which falls into the “time window” period (the first 3 weeks). One week later, the mice were then challenged for 5 days, and features of allergic inflammation reached their peak a day or two after the final exposure. When we measured the airway responses, the age of these sensitized and challenged mice was around 4 weeks old, which is about the age of early puberty in human. Therefore, at least in this study, the protective effects of LPS lasted till puberty. Further studies are needed to assess whether the protective effects will last longer and the underlying mechanisms.

To further elucidate the underlying mechanism of this age-dependent effects of LPS treatment, we evaluated the milieu of peritoneal cavity. We found that in homeostasis, the composition of immune cells in the peritoneal cavity varied greatly in different age groups. B cells accounted for the largest proportion in adult mice (about 60%), which is consistent with previous reports (22), while in neonates, peritoneal macrophages accounted for the largest proportion (about 70%). Inflammatory monocytes that were recruited to the peritoneal cavity following OVA/alum sensitization have been illustrated to play a key role in mediating the Th2 responses (21). In the current study, we observed a similar monocyte infiltration which peaked at 16 h. However, neonatal mice showed a much lower number of monocytes compared with the positive control upon LPS treatment, while in adult mice, LPS treatment only postponed the recruitment of monocytes. Subsequently, we compared the phenotype of these cells and found that the expression of CD80/CD86/OX40L on the inflammatory monocytes of neonatal mice was significantly higher than that of adult mice, especially OX40L. However, when monocytes from peripheral blood of both ages were compared, the expression of OX40L was low and there was no age difference (**Supplementary Figure 2C**). These data suggest that the phenotypic difference is not an intrinsic character of neonatal monocytes. It is the peritoneal milieu, which is highly permissive for Th2 cell differentiation by induction of a high expression of OX40L on monocytes, especially in neonatal mice after OVA/alum sensitization. This may partially explain the characteristics of type 2 responses induced by alum. Similar results have been found in our previous studies, that is, the expression of OX40L molecule on lung DCs of neonatal mice infected with RSV was significantly higher than that of the adult counterpart (30).

In this study, we found that LPS treatment had no significant effect on the expression of CD80/CD86/OX40L, except only that it increased the expression level of CD80. Regarding the cytokine expression profile of monocytes, we found that LPS treatment resulted in higher expression of IL-12 and IFN-γ in adult mice, which was consistent with previous reports (31), indicating a Th1 polarization. Furthermore, the increased expression of TGF-β and the increased level of IL-6 in the PLF in the adult group after LPS treatment indicated a tendency of Th17 polarization. In brief, LPS treatment in neonatal mice significantly reduced the number of monocytes, whereas in adult mice, LPS did not significantly affect the chemotaxis of monocytes but mainly changed the secretion profile of cytokines.

Eosinophils have been reported to be a major source of early IL-4 after alum injection (32, 33). OVA/alum sensitization induced the infiltration of eosinophils and LPS treatment significantly reduced the number of eosinophils in both age groups. Neutrophils are among the first-line innate responders in inflammation. Few studies have looked at their potential implications in allergic asthma (34). Recently, emerging evidence started to show that some neutrophil subsets are endowed with immunoregulatory properties in allergic asthma (35). In this study, we found that there were two waves of neutrophil infiltration. The first one was induced by LPS treatment and the second one was induced by alum. LPS treatment significantly increased the number of neutrophils in both age groups. Together, the decreased eosinophils and increased neutrophils in the peritoneal cavity upon LPS treatment further weakened the milieu for Th2 responses. Recent studies have shown that the adjuvant effect of alum may be caused by the release of danger signals. Consistent with previous studies (21, 24), uric acid and IL-33 were released shortly after OVA/alum sensitization in both age groups. However, LPS treatment had no effect even though it can reduce the type 2 responses. These results suggest that there are some factors induced by LPS that interfered with the effects of danger signals. We also tried to determine whether the cytokine milieu is biased toward Th1 or Th2 responses. We found that OVA/alum did not induce the expression of IL-12 and IFN-γ in both age groups, and only LPS-treated mice showed a transient and rapid increase. A small difference was found: in the neonatal group, the levels decreased to control levels at 24 h, whereas in the adult group, they were maintained at high levels at 24 h. Therefore, the protective effect of LPS may be related to Th1 polarization, especially in adult mice.

The most significant age-dependent difference of LPS treatment is the number of inflammatory monocytes. Given the decreased number of monocytes at each time point upon LPS treatment in neonates, we first assumed that it might be the recruitment of monocytes that was inhibited. We did observe the rapid increase of CCL-2 and CCL-5 following OVA/alum injection in both age groups, which is accompanied with the increased number of monocytes. This finding is in line with several reports showing that alum induces monocyte recruitment to the peritoneal cavity (21, 31, 33, 36, 37). To our surprise, LPS treatment resulted in slightly reduced levels of CCL-5 or even higher levels of CCL-2, which was contradictory to the observed decreased monocytes after LPS treatment in neonatal mice. There might be some possibilities. Firstly, there are chemokines, other than CCL-2 and CCL-5, which are responsible for monocyte migration in the neonates. Secondly, the elevated IL-6 was observed in the neonatal group. It has been recognized that monocyte recruitment into peripheral tissue is followed by their rapid differentiation toward either macrophages or DCs, which are crucial players in the innate response or adaptive immune responses, respectively. LPS or IL-6 has been shown to drive the differentiation of monocytes toward macrophage (38–40). Therefore, we speculated that although LPS treatment could recruit monocytes to the peritoneal cavity, these cells differentiate into peritoneal macrophages instead of DC, which would go through the “macrophage disappearance reaction (MDR)” (41, 42). Last but not the least, it has been reported recently that neutrophils are heterogeneous and there are suppressive neutrophils which can inhibit type 2 response induced by HDM or OVA (43). Neutrophils have also been shown to inhibit the HDM-induced type 2 response by inhibiting chemotaxis of inflammatory monocytes and antigen presentation of monocytes (44). Since the number of monocytes was much lower upon LPS treatment in the neonatal group, we speculated that LPS-induced neutrophils might inhibit the recruitment of monocytes.

In summary, our work highlights the age-dependent effects of LPS on the induction of Th2 allergic responses. Although the type 2 responses were ameliorated by LPS in both age groups, Th17 polarization and neutrophil infiltration were induced at the same time in adult mice. LPS treatment reduced the number of inflammatory monocytes in neonatal mice, while it shifted the function of these cells in the adult group. In both age groups, LPS preconditioning was associated with decreased eosinophils and increased neutrophils, further weakening the milieu for Th2 responses. Our findings provide new evidence that the immune response varies between ages. Further understanding of the mechanisms by which this occurs could lead to better strategy for intervention of childhood allergic asthma and for vaccine design in children.

## Data Availability Statement

The raw data supporting the conclusions of this article will be made available by the authors, without undue reservation.

## Ethics Statement

The animal study was reviewed and approved by the Animal Ethical Committee of Huazhong University of Science and Technology.

## Author Contributions

LG, YH, and JH designed the study and wrote the manuscript. LG, HL, MH, and RS performed the experiments. MW established the technique of FACS. LG analyzed the data. All authors contributed to the article and approved the submitted version.

## Funding

This work was supported by the National Natural Science Foundation of China (31770994 and 31970865 to JH).

## Conflict of Interest

The authors declare that the research was conducted in the absence of any commercial or financial relationships that could be construed as a potential conflict of interest.

## Publisher’s Note

All claims expressed in this article are solely those of the authors and do not necessarily represent those of their affiliated organizations, or those of the publisher, the editors and the reviewers. Any product that may be evaluated in this article, or claim that may be made by its manufacturer, is not guaranteed or endorsed by the publisher.

## Supplementary Material

Supplementary Figure 1 Gating strategy to identify immune cells in the lung and peritoneal cavity. **(A–D)** Single cell suspension from lung and peritoneal cavity were prepared and assessed by flow cytometry. **(A)** Lung eosinophils (Siglec-F^+^ CD64^-^). **(B)** Lung CD103^+^DCs (CD11c^+^MHCII^hi^CD103^+^CD11b^-^) and CD11b^+^DCs (CD11c^+^ MHCII^hi^ CD103^-^ CD11b^+^). **(C)** Peritoneal eosinophils (CD45^+^SSC^hi^ MHCII-Siglec-F^+^), neutrophils (CD45^+^SSC^hi^Siglec-F-Ly6G^+^ CD11b^+^), macrophages (CD45^+^SSC^hi^Siglec-F-Ly6G-F4/80^hi^CD11b^+^), B cells (CD45^+^SSC^low^CD19^+^) and T cells (CD45^+^SSC^low^CD3^+^). **(D)** Peritoneal inflammatory monocytes (Ly6C^hi^CD11b^+^). **(E)** The FMO controls and MFI of CD80, CD86 and OX40L on inflammatory monocytes mentioned in the Figure 3C-E. FSC, forward scatter; SSC, side scatter; FMO, fluorescence minus one; MFI, median fluorescence intensity.

Supplementary Figure 2 The expression of CD80, CD86 and OX40L on peripheral blood monocytes. Peripheral blood cells from naive mice of both ages were assessed by flow cytometry. **(A)** The gating strategy of blood monocyte. **(B)** The percentage of blood monocytes. **(C)** The expressions of CD80, CD86 and OX40L on blood monocytes. Data are representative of three independent experiments with *n* = 3 mice per group. Data are shown as mean ± SD. **P* < 0.05.
