# Supplementary figures and images for: Neonatal LPS Administered Before Sensitization Reduced the Number of Inflammatory Monocytes and Abrogated the Development of OVA-Induced Th2 Allergic Airway Inflammation
Source: Front Immunol. 2021 Sep 22;12:725906. doi: 10.3389/fimmu.2021.725906 (PMC8493091; doi:10.3389/fimmu.2021.725906)

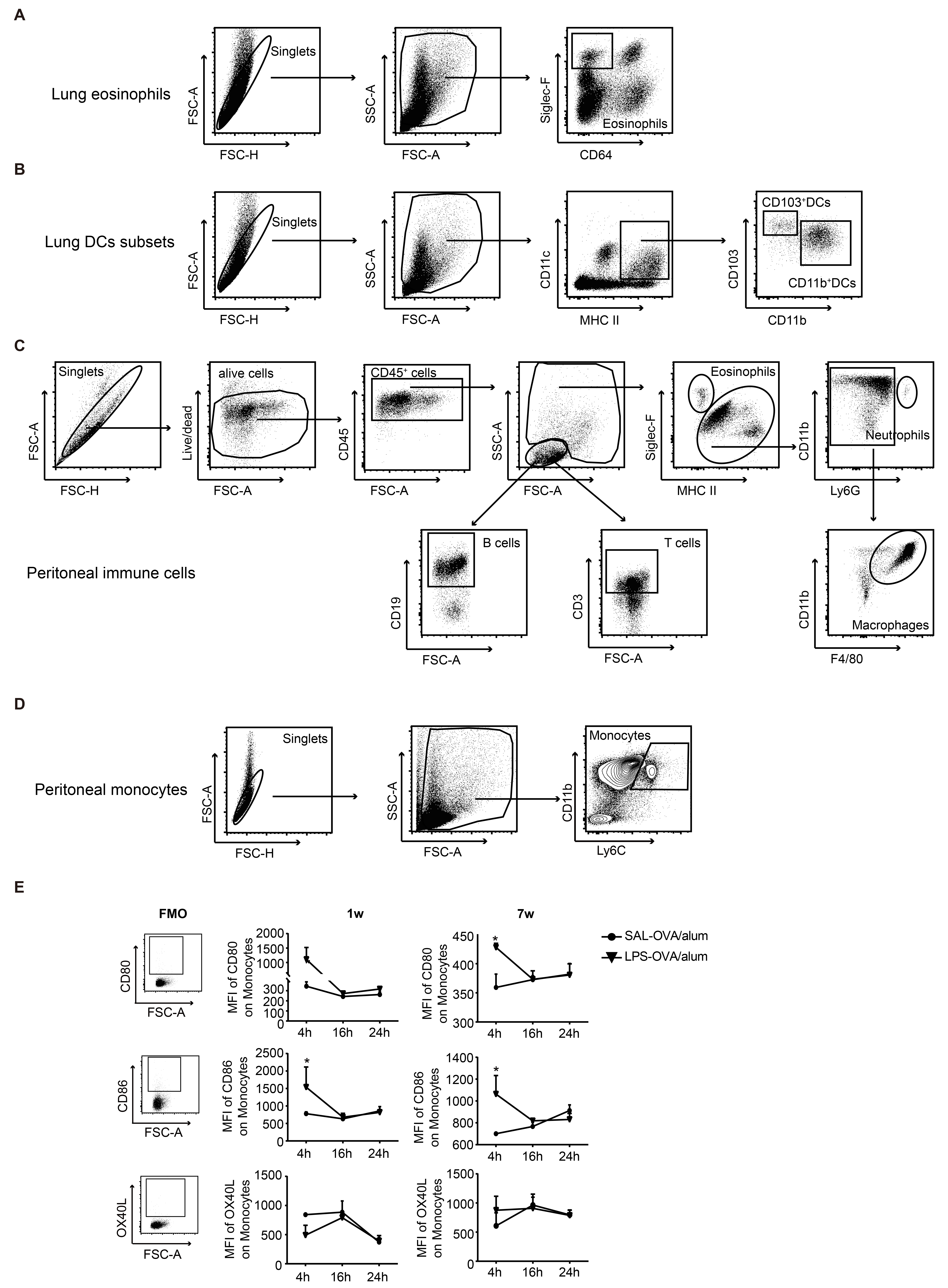

Supplement: Supplementary Figure 1 — Gating strategy to identify immune cells in the lung and peritoneal cavity. (A–D) Single cell suspension from lung and peritoneal cavity were prepared and assessed by flow cytometry. (A) Lung eosinophils (Siglec-F+ CD64-). (B) Lung CD103+DCs (CD11c+MHCIIhiCD103+CD11b-) and CD11b+DCs (CD11c+ MHCIIhi CD103- CD11b+). (C) Peritoneal eosinophils (CD45+SSChi MHCII-Siglec-F+), neutrophils (CD45+SSChiSiglec-F-Ly6G+ CD11b+), macrophages (CD45+SSChiSiglec-F-Ly6G-F4/80hiCD11b+), B cells (CD45+SSClowCD19+) and T cells (CD45+SSClowCD3+). (D) Peritoneal inflammatory monocytes (Ly6ChiCD11b+). (E) The FMO controls and MFI of CD80, CD86 and OX40L on inflammatory monocytes mentioned in the Figure 3C-E. FSC, forward scatter; SSC, side scatter; FMO, fluorescence minus one; MFI, median fluorescence intensity. [file Image_1.tif]

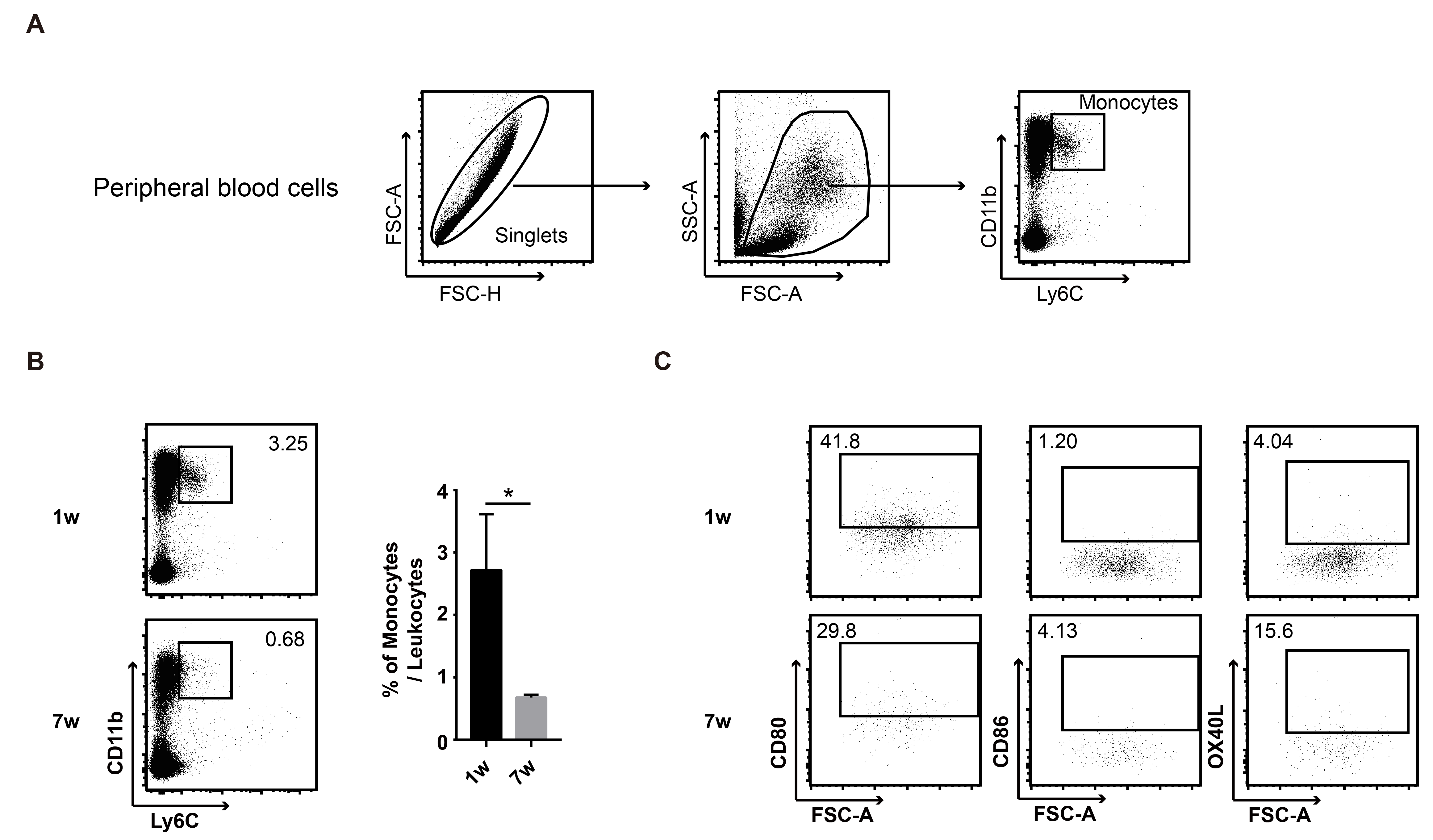

Supplement: Supplementary Figure 2 — The expression of CD80, CD86 and OX40L on peripheral blood monocytes. Peripheral blood cells from naive mice of both ages were assessed by flow cytometry. (A) The gating strategy of blood monocyte. (B) The percentage of blood monocytes. (C) The expressions of CD80, CD86 and OX40L on blood monocytes. Data are representative of three independent experiments with n = 3 mice per group. Data are shown as mean ± SD. *P < 0.05. [file Image_2.tif]
