# Supplementary material for: Neonatal LPS Administered Before Sensitization Reduced the Number of Inflammatory Monocytes and Abrogated the Development of OVA-Induced Th2 Allergic Airway Inflammation
Source: Front Immunol. 2021 Sep 22;12:725906. doi: 10.3389/fimmu.2021.725906 (PMC8493091; doi:10.3389/fimmu.2021.725906)
Supplement: Supplementary file 3 [file Table_1.pdf]

Supplementary Table 1. The primers used in this study were listed

Gene	sequence of Gene
β-actin-F	CGCAGCCACTGTCGAGTC
β-actin-R	CGCAGCGATATCGTCATCCAT
IL-6-F	AGCCAGAGTCCTTCAGAGAGAT
IL-6-R	AGGAGAGCATTGGAAATTGGGG
IL-1β-F	GCCACCTTTTGACAGTGATGAG
IL-1β-R	TGCTGCGAGATTTGAAGCTG
IL-10-F	AGGCGCTGTCATCGATTTCT
IL-10-R	ATGGCCTTGTAGACACCTTGG
TNF-α-F	CTAGTCCCTTGCTGTCCTCG
TNF-α-R	CCGAGGGTTGAATGAGAGCTT
TGF-β-F	ATGCTAAAGAGGTCACCCGC
TGF-β-R	TGCTTCCCGAATGTCTGACG
IL-12-F	CGCAGCACTTCAGAATCACA
IL-12-R	TCTCCCACAGGAGGTTTCTG
IFN-γ-F	TCAAGTGGCATAGATGTGGAAGAA
IFN-γ-R	TGGCTCTGCAGGATTTTCATG
